# Low-frequency vibrational modes of stable glasses

**DOI:** 10.1038/s41467-018-07978-1

**Published:** 2019-01-03

**Authors:** Lijin Wang, Andrea Ninarello, Pengfei Guan, Ludovic Berthier, Grzegorz Szamel, Elijah Flenner

**Affiliations:** 1 Beijing Computational Science Research Center, 100193 Beijing, China; 2 Department of Chemistry, Colorado State University, Fort Collins, CO 80523 USA; 3 Laboratoire Charles Coulomb (L2C), University of Montpellier, CNRS, 34095 Montpellier, France; 4 CNR-ISC, Uos Sapienza, Piazzale A. Moro 2, 00185 Roma, Italy

## Abstract

Unusual features of the vibrational density of states *D*(*ω*) of glasses allow one to rationalize their peculiar low-temperature properties. Simulational studies of *D*(*ω*) have been restricted to studying poorly annealed glasses that may not be relevant to experiments. Here we report on *D*(*ω*) of zero-temperature glasses with kinetic stabilities ranging from poorly annealed to ultrastable glasses. For all preparations, the low-frequency part of *D*(*ω*) splits between extended and quasi-localized modes. Extended modes exhibit a boson peak crossing over to Debye behavior (*D*_ex_(*ω*) ~ *ω*^2^) at low-frequency, with a strong correlation between the two regimes. Quasi-localized modes obey *D*_loc_(*ω*) ~ *ω*^4^, irrespective of the stability. The prefactor of this quartic law decreases with increasing stability, and the corresponding modes become more localized and sparser. Our work is the first numerical observation of quasi-localized modes in a regime relevant to experiments, and it establishes a direct connection between glasses’ stability and their soft vibrational modes

## Abstract (web summary)

The nature of the vibrational modes of amorphous solids is of fundamental interest, but assessing them is challenging due to very long equilibrium times involved. Wang et al. numerically model the localized low-frequency vibrational modes in glasses and show the sensitivity of their populations to glass stability.

## Introduction

Amorphous solids exhibit universal low-temperature properties, seen for instance in the heat capacity and thermal conductivity^1^, that differ remarkably from crystal physics. These properties are related to the vibrational density of states *D*(*ω*). For a continuous elastic medium in three dimensions, low-frequency excitations are phonons, and the density of states follows *D*(*ω*) = *A*_D_*ω*^2^, where *A*_D_ is given by Debye theory^2^. A well-known universal feature of amorphous solids is an excess in vibrational modes over the Debye prediction that results in a peak in *D*(*ω*)/*ω*^2^ at an intermediate frequency, called the boson peak^3–6^.

More recently, another source of ‘excess modes’ has been identified in computer simulations of model glasses^7–12^. It is composed of quasi-localized low-frequency modes with a density obeying *D*_loc_(*ω*) ~ *ω*^4^. Quasi-localized modes are observed at frequencies significantly lower than the boson peak and the link between the two phenomena is not immediate, despite some indications that they may be connected^8,13^. The quartic law was predicted long ago using phenomenological models^14,15^, reanalyzed over the years^16–18^, and remains the focus of intense research^19,20^. These predictions differ from two recent mean-field approaches^21,22^, which predict instead a universal non-Debye behavior that is quadratic in all spatial dimensions, also reported numerically^23^. Interest in the low-frequency localized modes extends beyond connections to theoretical models and the boson peak. It was suggested that these modes are correlated with irreversible structural relaxation in the supercooled liquid state^24^, and that the spatial distribution of these soft modes is correlated with rearrangements upon mechanical deformation and plasticity^25–28^. Localized defects are also central to theoretical descriptions of glass properties at cryogenic temperatures^29,30^.

Recent numerical insights were obtained for glasses that are very different from the ones studied experimentally, since they are prepared with protocols operating on timescales that differ from experimental ones by as many as ten orders of magnitude^31^. It is therefore unknown whether any of the vibrational, thermal, or mechanical properties derived from earlier computational study of the density of states is experimentally relevant. For example, it was reported^9,32^ that *D*_loc_(*ω*) ~ *ω*^*β*^ with *β* ranging from 3 to 4 depending on the glass’s stability, with *β* = 4 for the two most stable simulated glasses created by cooling at a constant rate. It remains unclear, however, whether *β* would be different for glasses with stability comparable to that of the experimental glasses.

Our main achievement is to extend studies of the vibrational density of states of computer glasses to an experimentally relevant regime of glass stability for the first time. To this end, we build on the recent development of a Monte Carlo method that allows us to equilibrate supercooled liquids down to temperatures below the experimental glass transition^33–35^ to prepare glasses that cover an unprecedented range of kinetic stability, from extremely poorly annealed systems to ultrastable glasses. We thus match the large gap between previous numerical findings and the experimental regime^36^. Recent studies have shown that that such stable glasses may differ qualitatively from ordinary computer glasses^35,37,38^. For example, qualitatively different yielding behavior of well-annealed glasses compared to that of poorly annealed glasses was reported in ref. ^38^. Since rearrangements upon mechanical deformation are correlated with the spatial distribution of soft modes, this result suggested that the density of states could also evolve dramatically with the stability.

## Results

### System preparation

We prepare glasses by instantaneously quenching supercooled liquids equilibrated at parent temperature *T*_p_ to *T* = 0, so that *T*_p_ uniquely controls the glass stability. We find that the low-frequency part of the vibrational density of states changes considerably when *T*_p_ varies, thus offering a direct link between soft vibrational modes and kinetic stability. Following earlier work^8,10^, we divide modes into extended and quasi-localized ones. As found for high parent temperature glasses^7–10^, the density of states of the quasi-localized modes follows *D*_loc_ = *A*_4_*ω*^4^, with the same quartic exponent for all glass stabilities. Our work thus establishes the relevance of earlier findings about quasi-localized modes and their effect on the density of states in the experimentally relevant regime of glass stability. In addition, we find that the overall scale *A*_4_ decreases surprisingly rapidly when *T*_p_ decreases, showing that the density of the quasi-localized modes is highly sensitive to the glass stability. This rapid decrease contrasts with the modest changes found for other structural quantities, such as mechanical moduli, sound speed, and Debye frequency. Quasi-localized modes also become sparser and increasingly localized at low *T*_p_, and so the identification of soft localized modes as relevant glassy defects controlling the physics of amorphous solids becomes more convincing near the experimental glass transition. Our results also suggest that ultrastable glasses contain significantly fewer localized excitations than ordinary glasses, which appears consistent with recent experiments^39–41^.

We simulate a polydisperse glass forming system in three dimensions, which is a representative glass-forming computer model^33^. We use the swap Monte Carlo algorithm to prepare independent equilibrated configurations at parent temperatures *T*_p_ ranging from above the onset temperature of slow dynamics *T*_o_ ≈ 0.200, down to *T*_p_ = 0.062, which is about 60% of the mode-coupling temperature *T*_c_ ≈ 0.108 (*T*_c_ marks a crossover to activated dynamics and corresponds typically to the lowest temperature accessed by standard molecular dynamics). Importantly, our lowest *T*_p_ is lower than the estimated experimental glass temperature *T*_g_ ≈ 0.072^33^, and no previous computational study has explored such range of glass stability. In addition, we also use a very high parent temperature which we refer to as *T*_p_ = ∞. We then probe vibrational properties of zero-temperature glasses produced by an instantaneous quench from equilibrated configurations at different *T*_p_. The specific simulation details are provided in Methods.

### Macroscopic properties

**Fig. 1:**
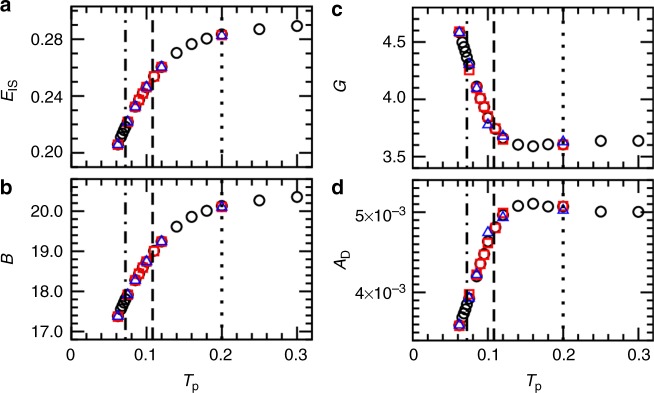
Macroscopic properties. **a** Inherent structure energy *E*_IS_; **b** Bulk modulus *B*; **c** Shear modulus *G*; **d** Debye level *A*_D_. In all panels, the vertical dashed-dotted, dashed, and dotted lines mark the positions of *T*_g_, *T*_c_, and *T*_o_, respectively. Data shown are for *N* = 48,000 (circles), 96,000 (squares), and 192,000 (triangles)

We begin by presenting macroscopic properties of the glasses as a function of the parent temperature *T*_p_. The inherent structure energy *E*_IS_ is directly related to the mobility of the particles^42^, and thus we show *E*_IS_ in Fig. 1a as an indicator of the increased stability of the glass. *E*_IS_ deviates from its high-temperature plateau when *T*_p_ becomes smaller than the onset temperature, and decreases further with decreasing *T*_p_^43^. Similarly, the bulk modulus *B* decreases modestly with decreasing *T*_p_ (Fig. 1b). By contrast, the shear modulus *G* in Fig. 1c remains nearly temperature-independent until the mode-coupling temperature, which is below the onset temperature, and then the shear modulus increases with decreasing *T*_p_. Associated with the increase in the shear modulus is a decrease in the Debye level $A_{\mathrm{D}} = 3/\omega _{\mathrm{D}}^3$, where the Debye frequency $\omega _{\mathrm{D}} = \left[ {\left( {18\pi ^2\rho } \right)/\left( {c_{\mathrm{l}}^{ - 3} + 2c_{\mathrm{t}}^{ - 3}} \right.} \right]^{1/3}$. The decrease of *A*_D_ is mainly controlled by the increase of the shear modulus since the transverse speed of sound $c_{\mathrm{t}} = \sqrt {G/\rho }$ is 2.4–2.6 times smaller than the longitudinal speed of sound *c*_l_. The overall relative variations of mechanical moduli and Debye frequency are, however, relatively mild given the broad range of glass stabilities covered in Fig. 1.

### Classification of quasi-localized and extended modes

**Fig. 2:**
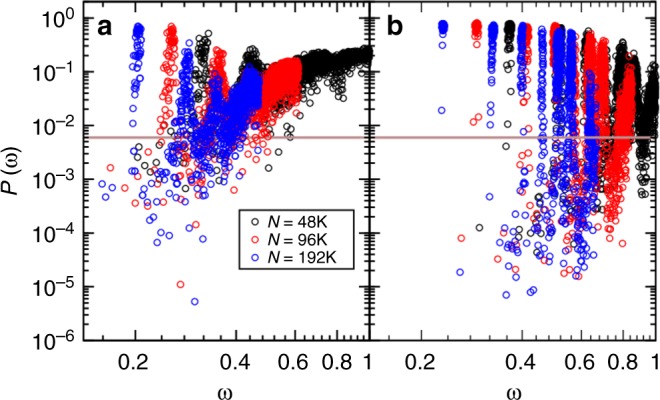
Classification of modes. Participation ratio *P*(*ω*) as a function of frequencies *ω* combined from systems with *N* = 48,000, 96,000, and 192,000 at parent temperatures *T*_p_ = 0.200 in (**a**) and *T*_p_ = 0.062 in (**b**). The horizontal line marks the threshold *P*_0_ = 0.006 between extended and quasi-localized modes

By examining the participation ratio *P*(*ω*) as a function of *ω* at different parent temperatures (see Fig. 2), we observe all the features that characterize the *T*_p_-dependence of the density of states. A value of *P*(*ω*) = 1 indicates a mode where all the particles participate equally, a value of *P*(*ω*) = *N*^−1^ indicates a mode where only one particle participates, and a value of *P*(*ω*) = 2/3 indicates a plane wave. The sharp peaks in *P*(*ω*) at low frequencies are due to the phonon modes, with the first peak corresponding to the first allowed transverse phonon at *ω*_t_ = *c*_t_2*π*/*L*, *L* being the box length. An increase in *ω*_t_ indicates an increase in $c_{\mathrm{t}} = \sqrt {G/\rho }$. The low-frequency modes can be naturally divided into quasi-localized modes (small *P*) and extended modes (large *P*) through an appropriate thresholding procedure^8,10^, this decomposition becoming sharper as *L* increases and *T*_p_ decreases. The value *P*_0_ = 0.006 is appropriate, as shown in Fig. 2, but we checked that our results are not qualitatively affected by a reasonable change of *P*_0_. As *T*_p_ decreases, phonon modes shift to larger frequencies, as expected from the evolution of the mechanical moduli, whereas quasi-localized modes become increasingly localized and well-separated from the phonons. We also checked that our results hold for small system sizes where allowed phonon modes are shifted to much higher frequencies^7^.

### Properties of quasi-localized modes

**Fig. 3:**
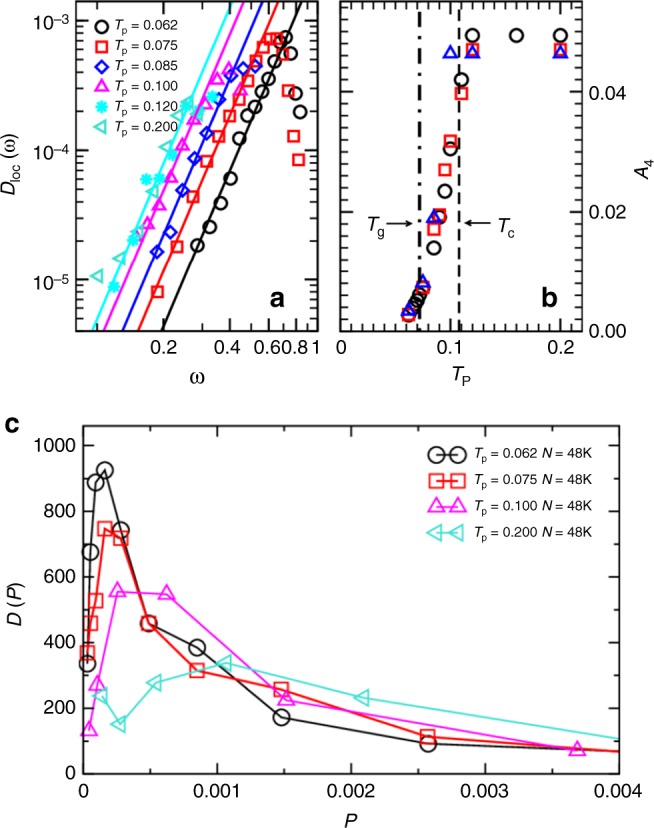
Density of states and spatial localization of quasi-localized modes. **a** Density of states *D*_loc_(*ω*) for quasi-localized modes for *N* = 48,000, with fits to *D*_loc_ = *A*_4_*ω*^4^. **b** *T*_p_ dependence of *A*_4_ for *N* = 48,000 (circles), 96,000 (squares), and 192,000 (triangles), with the mode-coupling temperature *T*_c_ and the estimated experimental glass temperature *T*_g_ indicated. **c** Probability distribution of the participation ratio for quasi-localized modes in the frequency-range of the *ω*^4^ scaling for various *T*_p_

We examined the density of states for the quasi-localized modes *D*_loc_(*ω*), which are shown in Fig. 3a for a few representative *T*_p_. At low frequencies, *D*_loc_(*ω*) = *A*_4_*ω*^4^ for each parent temperature with a prefactor *A*_4_ that depends on the glass stability. We show the resulting *A*_4_(*T*_p_) in Fig. 3b. The prefactor *A*_4_ stays nearly constant for high enough *T*_p_, but decreases sharply when *T*_p_ decreases below the mode-coupling temperature *T*_c_. This observation is robust against changing the system size. The decrease of *A*_4_ at low *T*_p_ correlates well with the evolution of shear modulus and Debye level in Fig. 3c, d. We note that a study of less stable glasses^32^ found an increase in the lowest frequency of quasi-localized modes with decreasing parent temperature, which, under certain assumptions, may be related to the change of *A*_4_ reported here. A major result of our study is that the quartic law governing *D*_loc_(*ω*) is obeyed irrespective of the glass stability, thus extending the validity of previous findings to the experimentally relevant regime.

In Fig. 3c we show the probability distribution for finding a mode with a participation ratio *P* for the modes with *P* < *P*_0_ for *N* = 48,000 particles. With decreasing *T*_p_, the distribution becomes narrower and the peak position shifts to smaller *P* values. We find that the average participation ratio decreases with decreasing *T*_p_, which is evident from Fig. 3c. This confirms that these modes become more localized with decreasing parent temperature, which had been observed for less stable glasses^9,13,32^. Since the density of states is a function of the structure of the quenched system, we conclude that subtle local structural changes occur for *T*_p_ below *T*_c_ that strongly affect soft vibrational motion in the quenched glass.

**Fig. 4:**
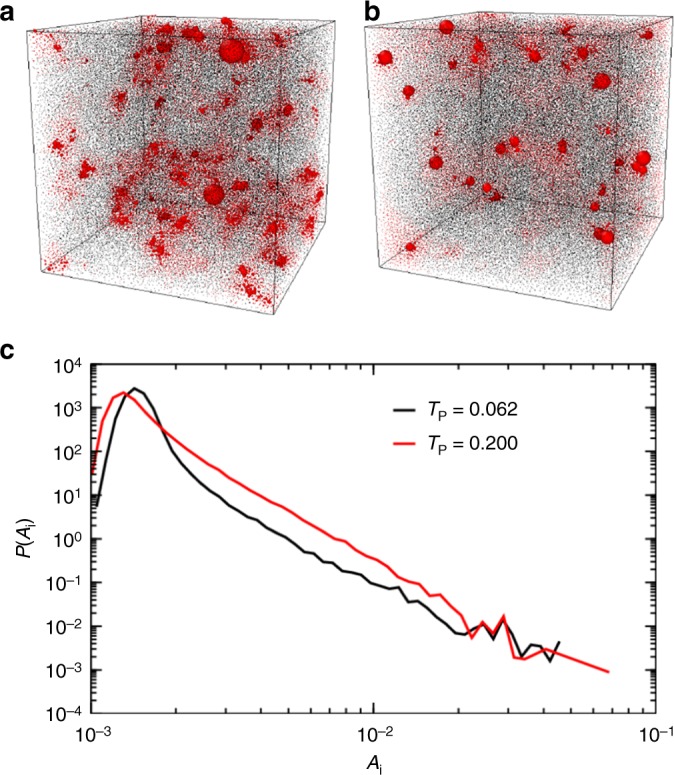
Softness of quasi-localized modes. Snapshots obtained for *T*_p_ = 0.200 (**a**) and *T*_p_ = 0.062 (**b**) with *N* = 192,000. The particles are shown with their radius given by the vibrational amplitude *A*(*i*) calculated from the lowest quasi-localized modes. **c** The probability distribution of *A*(*i*) for *T*_p_ = 0.200 and *T*_p_ = 0.062. For lower parent temperatures there is a smaller fraction of the particles with larger *A*(*i*), and thus the modes are more localized

To visualize the increasing mode localization, we define a ‘softness’^25^ for particle *i* as $A(i) = (1/M)\mathop {\sum}\nolimits_{l = 1}^M |{\mathbf{e}}_{l,i}|$, where the sum is taken over the *M* = 40 lowest frequency quasi-localized modes for one inherent structure (we have checked that our conclusions hold when we take other values of *M* = 5–40). The softness quantifies the vibrational amplitude of low-frequency quasi-localized modes. In the snapshots of Fig. 4, particles are represented with a size proportional to *A*(*i*) for (a) *T*_p_ = 0.200 and (b) *T*_p_ = 0.062. For the highest *T*_p_, clusters contributing to localized modes are relatively numerous, quite extended, and strongly coupled to their environment. At the lowest *T*_p_, each cluster is localized around just a few particles, there are much fewer clusters, and they offer a stronger contrast with the immobile background. To quantify these observations, we measured the probability distribution of *A*(*i*) (Fig. 4c). These distributions show a power-law tail at large *A* values, $P(A_i) = \lambda (T_{\mathrm{p}})A_i^{ - \alpha }$ with *α* ≈ 3.7. At low *T*_p_ the tail is well separated from the core of the distribution at small *A*, and mobile particles with large *A* are better defined. There is also a pronounced decay of the probability of finding large *A* values at low *T*_p_ since *λ*(0.2)/*λ*(0.062) ≈ 4.3, which indicates a greater than four fold decrease in the number of soft particles with large vibrational amplitudes. The interpretation of quasi-localized modes as relevant glassy defects controlling mechanical and thermal properties of glasses is therefore more convincing for stable glasses than it is for conventional computer glasses.

### Properties of extended modes

**Fig. 5:**
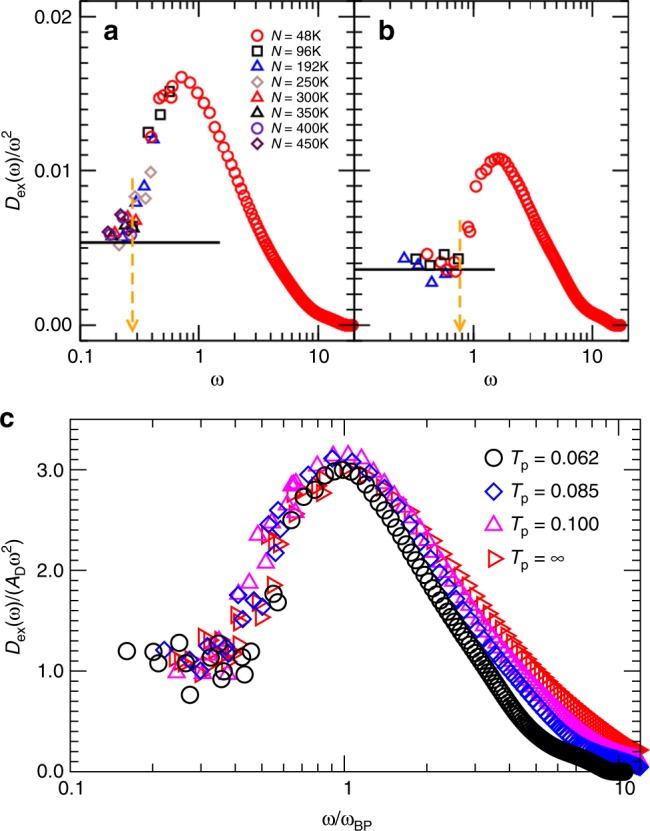
Density of states for extended modes. Reduced density of states for extended modes, *D*_ex_(*ω*)/*ω*^2^, at parent temperatures *T*_p_ = ∞ (**a**) and 0.062 (**b**), for systems with different sizes. The black line in each panel indicates the Debye level *A*_D_ while the vertical arrow marks the frequency, where *D*_ex_(*ω*)/*ω*^2^ starts to deviate from *A*_D_. **c** Rescaled version of the same data using the Debye level (vertical axis) and the boson peak frequency *ω*_BP_ (horizontal axis)

Next, we examine the density of states of extended modes, *D*_ex_(*ω*), with a participation ratio greater than *P*_0_. In Fig. 5a, b we show the reduced density of states *D*_ex_(*ω*)/*ω*^2^ for two parent temperatures. For each temperature, the Debye level is reached at low enough *ω* and a boson peak is observed at larger frequencies. Using our localization criterion, we find that modes near the boson peak are not localized, but this does not imply that they have a phononic character. The boson peak narrows slightly with decreasing *T*_p_. The Debye level, the boson peak location, height, and width all change modestly as *T*_p_ is varied over the entire range studied. The changes observed in our study agree qualitatively with those found by Grigera et al. ^4^.

In Fig. 5c we examine scaling properties of the density of states of extended modes. We rescale *ω* by the boson peak frequency, *ω*_BP_, and plot the rescaled density of states *D*_ex_/(*A*_D_*ω*^2^). We observe an excellent collapse on the low-frequency side of the boson peak. This shows that in this frequency range the reduced frequency dependence has a universal shape, as reported before^44^. Second, the collapse also shows that the height of the boson peak correlates with the Debye level *A*_D_. These results agree with experiments on molecular glass formers^45–47^. However, some of the same experiments report that the boson peak position scales as the Debye frequency^45,47^, which is not consistent with our results. We also find that a scaling of *ω*_BP_ with the bulk modulus suggested in ref. ^48^ is inconsistent with our results. Note that we study the evolution of the boson peak as a function of the preparation temperature, while experiments sometimes examine the temperature evolution of the boson peak for a given glass preparation. We also note that a correlation between the boson peak and quasi-localized modes has been proposed by studying systems at different pressures around the unjamming transition^49^.

**Fig. 6:**
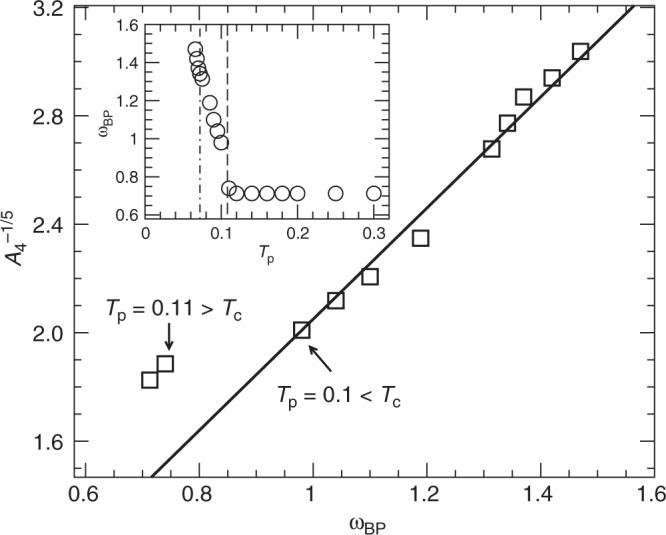
Scaling of the boson peak frequency. The characteristic frequency $A_4^{ - 1/5}$ versus the boson peak position *ω*_BP_. The line is a fit $A_4^{ - 1/5}\sim \omega _{{\mathrm{BP}}}$ for glasses whose *T*_p_ < *T*_c_, which is the parent temperature range where we see an increase in *ω*_BP_ with decreasing *T*_p_, see inset where the vertical dashed-dotted, dashed lines mark the positions of *T*_g_ and *T*_c_, respectively

Since the boson peak occurs in a different frequency range than the *ω*^4^ scaling of *D*_loc_(*ω*), it is not clear that there could be a relationship between the boson peak and the low-frequency quasi-localized modes. Simulations close to jamming suggest that $A_4\sim \omega _{{\mathrm{BP}}}^4$^8^, but we do not find that this relation holds with changing *T*_p_. An alternative possibility can be obtained from dimensional analysis, where a characteristic frequency for quasi-localized modes can be defined as $A_4^{ - 1/5}$^32^. We find that $A_4^{ - 1/5}\sim \omega _{{\mathrm{BP}}}$ for glasses with *T*_p_ < *T*_c_ (Fig. 6), but this relation does not hold for glasses created with *T*_p_ > *T*_c_. We note that *ω*_BP_ is constant for *T*_p_ > *T*_c_, see the inset to Fig. 6, and only changes for *T*_p_ < *T*_c_. Again we find that *T*_c_ marks a change in the behavior of *D*(*ω*). Given the relatively small changes in both *ω*_BP_ and $A_4^{ - 1/5}$ over our entire range of parent temperatures studied, it is not clear that a power law is the proper relationship between these quantities and further work is needed to verify it.

## Discussion

In summary, we report the first characterization of the vibrational density of states of computer glasses prepared over a range of glass stability that bridges the gap between ordinary simulations and experimental studies. At low-frequency extended and quasi-localized modes coexist, and both types of modes evolve differently when the glass stability is varied. We find a relatively mild temperature dependence of extended modes, with a strong correlation between the Debye level and the boson peak. By contrast, quasi-localized modes evolve more strongly when *T*_p_ decreases below the mode-coupling temperature, but their density of states is always described by $D_{{\mathrm{loc}}}\sim A_4\omega ^4$. Unexpectedly, the temperature dependence of the prefactor *A*_4_(*T*_p_) is more interesting than the value of the quartic exponent, which is insensitive to the degree of annealing.

The increasing localization of the modes implies that subtle yet significant changes occur in the local structure of the glass that are not reflected in the pair correlation function, which is nearly identical for parent temperatures below *T*_c_. Since soft modes have been linked to irreversible relaxation^24^ and rearrangements under shear^25–28^, it follows that the reduction of these soft modes can have significant implications for glassy dynamics. In turn this reduction indicates that there are fewer soft spots, which should increase the strength of the glass. This hypothesis is supported by the observation that the decrease in *D*_loc_(*ω*) mirrors the increase of the shear modulus, and also correlates very well with the evolution of the ductility of the produced glasses^38,50^. Since we can now equilibrate amorphous systems at temperatures low enough so that they do not flow, another perspective would be to analyze the density of states at finite temperatures through the Fourier transform of the velocity autocorrelation function^51^, or by diagonalizing the covariance matrix of displacements^52^. Future studies should examine the difference between these procedures to provide insights into thermal anharmonicities of stable glasses, and more generally into their low-temperature transport properties.

## Methods

### Simulations

We simulate a polydisperse model glass former of sizes between *N* = 48,000 and 450,000 particles with equal mass at a number density *ρ* = 1.0^33^. The interaction between two particles *i* and *j* is given by $V(r_{ij}) = \left( {\frac{{\sigma _{ij}}}{{r_{ij}}}} \right)^{12} + v(r_{ij})$ when their separation $r_{ij} \le r_{ij}^c = 1.25\sigma _{ij}$ and zero otherwise. We use $v(r_{ij}) = c_0 + c_2\left( {\frac{{r_{ij}}}{{\sigma _{ij}}}} \right)^2 + c_4\left( {\frac{{r_{ij}}}{{\sigma _{ij}}}} \right)^4$, where the coefficients *c*_0_, *c*_2_, and *c*_4_ ensure the continuity of *V*(*r*_*ij*_) up to the second derivative at the cutoff $r_{ij}^c$. The probability of particle diameters *σ* is *P*(*σ*) = *A*/*σ*^3^, where *σ*∈[0.73,1.63] and we use a non-additive mixing rule, $\sigma _{ij} = \frac{{\sigma _i \,+\, \sigma _j}}{2}(1 - 0.2|\sigma _i - \sigma _j|)$. For *N* ≤ 192,000 we use the swap Monte Carlo algorithm to prepare independent equilibrated configurations at parent temperatures *T*_p_ ranging from above the onset temperature of slow dynamics (*T*_o_ ≈ 0.200) down to *T*_p_ = 0.062, which is about 60% of the mode-coupling temperature (*T*_c_ ≈ 0.108), and is lower than the estimated experimental glass temperature (*T*_g_ ≈ 0.072)^33^. In addition, we also use a very high parent temperature, which we refer to as *T*_p_ = ∞. Due to very long equilibration times for systems of *N* > 192,000 particles we only study systems with *N* > 192,000 for *T*_p_ = ∞.

### Density of states calculation

Following equilibration at a temperature *T*_p_, zero-temperature glasses are produced by instantaneously quenching equilibrium configurations to their inherent structures using the Fast Inertia Relaxation Engine algorithm^53^. We then calculate the modes by diagonalizing the Hessian matrix using Intel Math Kernel Library (https://software.intel.com/en-us/mkl/) and ARPACK (http://www.caam.rice.edu/software/ARPACK/). We calculate all the normal modes for the 48,000 particle systems, but only the low-frequency part of the spectrum in systems with *N* > 48,000. We characterize the modes through the density of states $D(\omega ) = \frac{1}{{3N - 3}}\mathop {\sum}\nolimits_{l = 1}^{3N - 3} \delta (\omega - \omega _l)$ and the participation ratio $P(\omega _l) = \frac{{\left( {\mathop {\sum}\nolimits_{i = 1}^N |{\mathbf{e}}_{l,i}|^2} \right)^2}}{{N\mathop {\sum}\nolimits_{i = 1}^N |{\mathbf{e}}_{l,i}|^4}}$, where ***e***_*l*,*i*_ is the polarization vector of particle *i* in mode *l* with frequency *ω*_*l*_. For a mode localized to one particle *P*(*ω*) = *N*^−1^, and for an ideal plane wave *P*(*ω*) = 2/3. The phonon modes occur at discrete frequencies, and care has to be taken in the binning procedure to calculate the density of states of extended modes, *D*_ex_(*ω*). To perform this calculation, we determine the phonon frequencies from the peak positions of the participation ratio versus frequency, and tune the bin size to smooth *D*_ex_(*ω*).To obtain the shear modulus *G* and the bulk modulus *B* we use the method described in ref. ^54^.

## Data Availability

All data will be available from the authors upon request.
